# Effects of the interaction between TiO_2_ with different percentages of exposed {001} facets and Cu^2+^ on biotoxicity in *Daphnia magna*

**DOI:** 10.1038/srep11121

**Published:** 2015-08-05

**Authors:** Lingling Liu, Wenhong Fan, Huiting Lu, Wei Xiao

**Affiliations:** 1 Department of Environmental Science and Engineering, School of Chemistry and Environment, Beihang University, Beijing 100191, Beijing, P. R.China; 2 Department of Environmental Engineering, School of Resource & Environmental Sciences, Wuhan University, Wuhan 430072, Hubei, P. R. China

## Abstract

Anatase TiO_2_ nanosheets (NSs) with exposed {001} facets have been widely used because of their high activity and particular surface atomic configuration. However, investigations on their biotoxicity are rare. In this study, bioaccumulation of five different TiO_2_ (with 10%, 61%, 71%, 74% and 78% exposed {001} facets), as well as copper and enzyme activities in *Daphnia magna*, are systematically investigated and rationalized. The results indicated that the addition of Cu^2+^ enhanced agglomeration–sedimentation of TiO_2_, resulting in the reduction of TiO_2_ bioaccumulation by 10% to 26%. TiO_2_ nanoparticles (NPs) increased copper bioaccumulation by 9.8%, whereas the other four TiO_2_ nanosheets (NSs) decreased it by 43% to 53%, which depended on TiO_2_ variant adsorption and free Cu^2+^ concentrations in the supernatant. The levels of superoxide dismutase (SOD) enzyme and Na^+^/K^+^-ATPase activities suggested that oxidative stress, instead of membrane damage, was the main toxicity in *D. magna*. Meanwhile, the SOD enzyme activities increased with decreasing Cu accumulation and increasing Ti accumulation because of the different functions of Cu and Ti in organisms. This research highlighted the important role of the percentage of exposed {001} facets in nanostructured TiO_2_ on bioaccumulation and biotoxicity of TiO_2_ and Cu^2+^ in *Daphnia magna*.

Nanomaterials are widely applied in various fields because of their unique physical and chemical properties. As one of the most commonly used nanomaterials, nano-sized TiO_2_ are widely used in photocatalysis, cosmetics, paint, medicine, and others. The estimated worldwide productions of nano-sized TiO_2_ are 2.5 million metric tons per year by 2025, which become a trillion US-dollar business in the future^1^,^2^.

The rapid expansion of nano-sized TiO_2_ increases the risk of aquatic environment exposure, which draws increasing attention. Lovern and Klaper first reported that nano-sized TiO_2_ was a hazardous material in aquatic organisms and the lethal concentration was only 10 ppm for *Daphnia magna* (*D.magna*) upon 48 h aqueous exposure^3^. However, the actual concentration of nano-sized TiO_2_ in natural water is very low (3 ng L^−1^ to 1.6 mg L^−1^), and reaching the lethal concentration is difficult^4^. Due to the special physicochemical characteristics of nano-sized TiO_2_, the existence of trace nano-sized TiO_2_ would influence the toxicity of original pollutants in the environment. Nano-sized TiO_2_ can adsorb other substances in water and influence their biological behaviors and toxicities. The presence of 2 mg L^−1^ nano-sized TiO_2_ increased the toxicity of the highly toxic marine antifouling compound tributyltin (TBT) up to 20-fold compared with TBT alone in abalone embryos^5^. For heavy metals, it was reported that the presence of TiO_2_ nanoparticles (NPs) as carriers greatly enhanced the accumulation of Cd and As in carp, and Cu in *D. magna*^6^,^7^. On the contrary, Yang’s research showed that nano-sized TiO_2_ diminished Cd^2+^ bioavailability and toxicity due to Cd^2+^ adsorption by TiO_2_, which decreased its ambient free ion concentrations^8^.

The biological toxicity of nano-sized TiO_2_ is closely related to its physicochemical characteristics, such as size, crystal and surface modifications, and radical formation. It was reported that anatase nano-sized TiO_2_ was more toxic than rutile, and NPs were more toxic than microparticles for cladocerans, algae, rotifers, and plants^9^. It was reported that biological surface coating of nano-sized TiO_2_ exerted a negative effect in the molting and development of *D. magna*^10^. The influence of TiO_2_ particle size on cadmium toxicity was confirmed, and Cd^2+^ with 30 nm TiO_2_ NPs presented more serious growth inhibition to algal^11^.

Recent research on nanostructured TiO_2_ focused on tailoring its shape, size, and exposed facets for enhancing its performance in photocatalysis, solar energy conversion, photochromic devices, and sensors, which was highlighted by the anomalous physicochemical properties of anatase TiO_2_ nanomaterials with different exposed {001} facets^12^,^13^. Theoretical and experimental studies have indicated that the {001} surface of anatase TiO_2_ is much more reactive than the thermodynamically more stable {101} surface because the average surface energy of the {001} facets of anatase TiO_2_ (0.90 J m^−2^) are twice higher than that of the {101} facets (0.44 J m^−2^)^14^. TiO_2_ nanosheets (NSs) with {001} facets exhibit high photocatalytic activity, and their photoactivity exceeds that of P25 by a factor of more than nine times^15^. However, the effects of TiO_2_ NSs with different exposed {001} facets on heavy metal accumulation and toxicity remain unexplored.

In this research, TiO_2_ NPs and NSs with different percentages of exposed {001} facets were prepared and characterized. Bioaccumulation of TiO_2_ and Cu^2+^ was investigated under different exposure conditions with *D. magna* as test organism. The changes in metabolic enzymes, such as superoxide dismutase (SOD) and Na^+^/K^+^-ATPase, were also discussed. The results of the present study provide a strong evidence for the environmental risks of TiO_2_ NPs and NSs.

## Results and Discussion

### Characterization of prepared TiO_2_

TiO_2_ samples with different percentages of exposed {001} facets were synthesized by changing ***R***_F_ and their physical properties are shown in Table 1. The percentage of the exposed {001} facet of TiO_2_ was calculated using the reported method according to crystal structure^16^. All the prepared TiO_2_ was anatase phase, according to X-ray diffraction results (not shown here). The BET surface areas of these TiO_2_ samples decreased from 156 m^2^ g^−1^ to 97 m^2^ g^−1^ with increasing {001} facet percentage from 10% to 78%. At the same time, the porosity of TiO_2_ increased from 55.0% to 67.5%, the pore volumes increased from 0.33 cm^3^ g^−1^ to 0.56 cm^3^ g^−1^, and the average pore size from 7.4 nm to 20 nm. The existing nanopores (or porosity) were from the aggregation of TiO_2_ NPs and NSs^17^.

**Figure 1:**
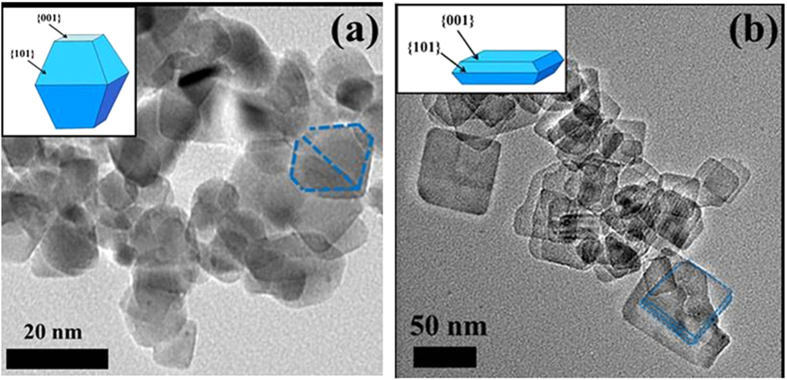
TEM images of the NP10 (**a**) and NS78 (**b**) samples.

TiO_2_ NPs (NP10) and NSs (NS78) were characterized by TEM, as shown in Fig. 1. The morphology of TiO_2_ NSs with 78% {001} facets (Fig. 1b) was different from that of TiO_2_ NPs with 10% {001} facets (Fig. 1a). According to Table 1, the shape of TiO_2_ changed from NPs to NSs with increasing *R*_F_. TiO_2_ with 61%, 71%, 74%, and 78% {001} facets were nanosheets, whereas those with 10% {001} facets were nanoparticles. The morphologies of TiO_2_ NS61, NS71, and NS74 were similar to that of NS78 (TEM pictures not shown).

### Adsorption of Cu^2+^ on TiO_2_

**Figure 2:**
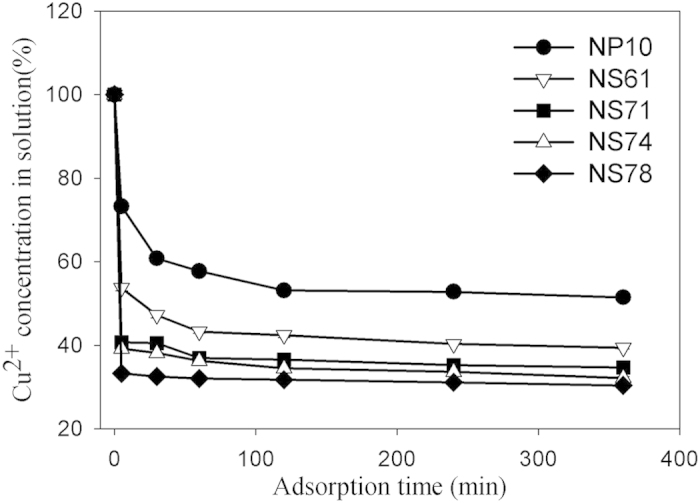
Cu^2+^ Adsorption on the prepared NP10, NS61, NS71, NS74 and NS78 samples in 500 mL of 1 mg/L TiO_2_ suspension solution. Mean ± standard deviation (n = 2).

The adsorption of Cu^2+^ on TiO_2_ was evaluated from the decrease of Cu^2+^ concentrations in the supernatant. Figure 2 shows rapid adsorption of Cu^2+^ on TiO_2_ with the adsorption equilibrium reaching within the first 60 min. The decrease on Cu^2+^ concentrations ranges from 50% to 70%, dependent on the percentage of exposed {001} facets in the samples. The TiO_2_ NSs with higher exposed {001} facets could adsorb more Cu^2+^ in water than the TiO_2_ NPs. This finding is related to the surface properties of {001} facets. The high-energy {001} facets of anatase TiO_2_ have more surface defects such as unsaturated Ti atoms and abundant oxygen holes that are more effective for the dissociative adsorption of H_2_O molecules than the thermodynamically more stable {101} facets^18^. As a result, a large number of OH groups are generated on {001} facets. Adsorbates tend to be adsorbed at steps, defects, and domain boundaries because the surface atoms at these sites have fewer coordination numbers^19^. Consequently, the neutral and unoccupied surface sites of TiO_2_ {001} facet are Ti-(OH)(OH_2_) in water^20^. (These surface OH groups participate in.) These surface OH groups have been proved to produce extra-active centers not only for small organic molecules adsorption^21^, but also metal ion adsorption on TiO_2_^22^. The exchange reaction of the metal cations with the -OH on the surface was presumed as presented in the following equation: TiOH^3+^ + Cu^2+^ → TiOCu^4+^ + H^+^. Therefore, the adsorption capacity of Cu^2+^ on TiO_2_ depends on the amount of OH groups on the TiO_2_ {001} surface. Hence, it is obvious that TiO_2_ NSs with 78% exposed {001} facets could adsorb the most Cu^2+^ in water.

### Accumulation of TiO_2_ in *D. magna*

**Figure 3:**
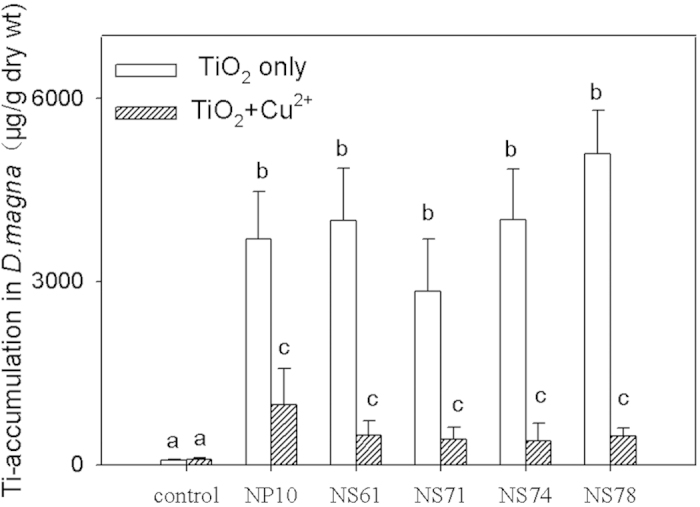
Accumulated Ti after 48 h exposure to 1 mg/L of the prepared NP10, NS61, NS71, NS74 and NS78 samples with or without 50 μg/L Cu^2+^. Mean ± standard deviation (n = 3), (*P* *<* 0.05, one-way ANOVA).

Ti accumulation in *D. magna* was determined after exposure to different TiO_2_ samples at the 1 mg L^−1^ concentration with and without Cu^2+^. As shown in Fig. 3, Ti accumulation in *D. magna* in the presence of Cu^2+^ was lower than that without Cu^2+^, suggesting that Cu^2+^ inhibited the ingestion of TiO_2_ by *D. magna*. When TiO_2_ and Cu^2+^ coexisted, Ti accumulation in *D. magna* exposed to the NP10 sample decreased by 26.4%. However, Ti accumulation in *D. magna* exposed to the other four NS samples (NS61, NS71, NS74, and NS78) decreased by 10%. Moreover, the accumulated Ti in *D. magna* increased slightly with increasing percentage of {001} facet of TiO_2_ NSs from 3692 μg g^−1^ (in NS 61) to 5088 μg g^−1^ (in NS 78) dry weight in the absence of Cu^2+^, except for the NS71 sample. Thus, the coexistence of Cu^2+^ and TiO_2_ NSs has a negative effect on bioaccumulation of TiO_2_ in organisms.

Free nanomaterials tend to aggregate in aquatic environments because of their large specific surface area. In addition, the less-mobile aggregated nanomaterials can easily combine with filter feeders and sediment-dwelling animals^23^. The aggregation is influenced by factors such as primary size, pH and ionic strength in aquatic environment^24^. According to the results of dynamic light scattering, these nano-sized TiO_2_ aggregated in water were from 1.363 μm to 1.572 μm in size in water with the absence of Cu^2+^, which further grew to about 2.1 μm in size when Cu^2+^was adsorbed onto the TiO_2_ surface. The addition of Cu^2+^ facilitates the aggregation of TiO_2_, in agreement of the reported increases the aggregation level when BPA was added into nano-TiO_2_ dispersions^25^. Dudev also demonstrated that the hydrodynamic diameter of anatase TiO_2_ nanoparticles (ANTNPs) increased in the presence of Ca^2+^, resulting in the aggregation of ANTNPs^26^. When the aggregated particle size exceeds a certain limit, the settlement behavior would become the key factor. The agglomeration– sedimentation processes resulted in the decreased concentrations of the NPs in the supernatant and then diminished the bioavailability of NPs^27^. The aggregation of nano-sized TiO_2_ has an important function in the environmental effects of NPs because the size and shape of NPs will determine the magnitude of any potentially toxic effect. In this experiment, Cu^2+^ enhanced the aggregation of TiO_2_ and formed the bigger aggregate in water, retarding the effective uptake of these particles by *D. magna*. Therefore, the existence of Cu^2+^ predictably weakened the bioaccumulation of nano-TiO_2_.

### Accumulation of copper in *D. magna*

**Figure 4:**
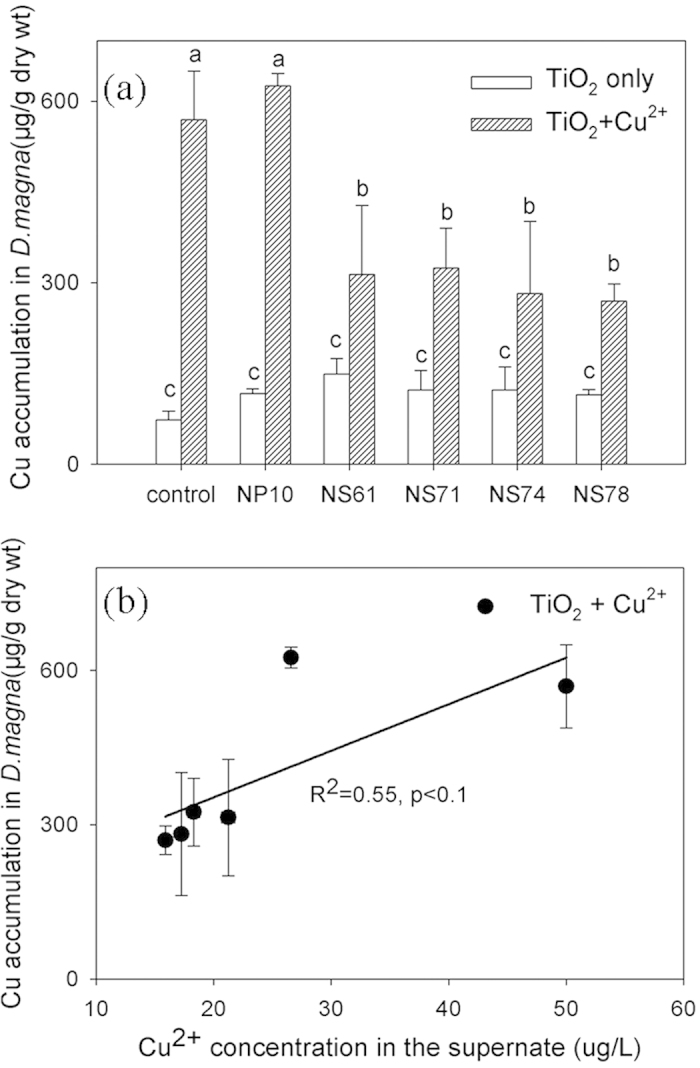
(**a**) Accumulated copper after 48 h exposure to 50 μg/L Cu^2+^ with or without 1 mg/L of the prepared NP10, NS61, NS71, NS74 and NS78 samples (*P* < 0.05, one-way ANOVA). (**b**) Relationship of copper accumulation in *D. magna* and Cu^2+^ concentration in the supernate when Cu^2+^ and TiO_2_ coexisted and reached a steady state in water. Mean ± standard deviation (n = 3).

Cu accumulation in *D. magna* at different exposure conditions was investigated in this study, as shown in Fig. 4a. Compared with the control experimental run (treated only with Cu^2+^), the existence of TiO_2_ also influenced the bioaccumulation of Cu^2+^ in *D. magna*. When *D. magna* was exposed to water with a mixture of Cu^2+^ and NP10, Cu^2+^ accumulation was enhanced by 9.8%. However, Cu^2+^ accumulation in *D. magna* was reduced by 43% to 53% when Cu^2+^ coexisted with TiO_2_ NSs. Generally, the forms of Cu^2+^ ingested by *D. magna* were free Cu^2+^ and adsorbed Cu^2+^ on TiO_2_. When copper coexisted with TiO_2_, TiO_2_ could adsorb Cu^2+^. Thus its free ion concentration decreased in the ambient environment, which diminished a portion of Cu^2+^ internalization and bioavailability. In contrast, Cu^2+^ accumulation was enhanced when *D. magna* swallowed Cu^2+^-adsorbed TiO_2_. The factor that dominates in Cu accumulation depends on the unique physicochemical characteristics of TiO_2_ and exposure condition.

The observed increase in copper accumulation with the presence of TiO_2_ NP10 is similar to previous report regarding the P25^28^. The explanation for reduced Cu accumulation with the presence of TiO_2_ NSs is as follows. Firstly, the Cu^2+^-adsorption capacities of TiO_2_ NSs were larger than those of TiO_2_ NPs, leading to the decrease in Cu^2+^ concentration. Yang studied Cd^2+^ toxicity caused by TiO_2_ NPs, and the results are similar to those of the present study. They suggested that nano-sized TiO_2_ could reduce free Cd^2+^ concentration in the media, which further lowers its bioavailability and toxicity to green alga *Chlamydomonas reinhardtii*^8^,^29^. As shown in Fig. 4b, a relative positive relationship exists between Cu accumulation in *D. magna* and Cu ion concentration in the media. The decrease of free Cu ion concentration was the main factor for the decrease of copper accumulation. Secondly, when Cu^2+^ coexisted with nano-sized TiO_2_, nano-sized TiO_2_ adsorbed Cu^2+^ and formed big aggregates in water. The large agglomeration–sedimentation of nano-sized TiO_2_ reduced Ti accumulation in *D. magna* and weakened the role of Cu as carrier. Thirdly, TiO_2_ NSs themselves may be toxic because of their insolubility in the gut and could alter Cu^2+^ toxicity in an antagonistic, synergistic, or additive way.

### SOD enzyme and Na^+^/K^+^-ATPase activity in *D. magna*

**Figure 5:**
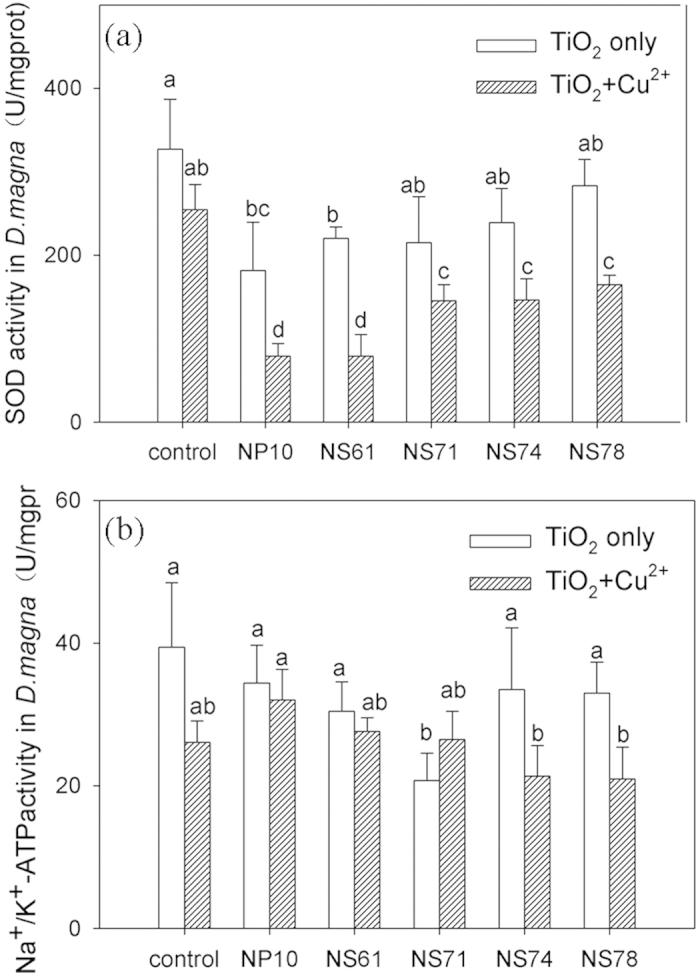
SOD enzyme (**a**) and Na^+^/K^+^-ATPase (**b**) activities in *D. magna* after 48 h exposure to the prepared NP10, NS61, NS71, NS74 and NS78 samples in the absence and presence of Cu^2+^. Mean ± standard deviation (n = 3), (*P* < 0.05, one-way ANOVA).

The SOD enzyme activities in *D. magna* were investigated because they are antioxidant biomarkers for oxidative stress. As shown in Fig. 5a, when *D. magna* was exposed only to different nano-sized TiO_2_, the SOD enzyme activity decreased from 55.5% to 86.6% compared with the control experiment. SOD enzyme activities increased with increasing percentage of {001} facet of TiO_2_ NSs, although the NS78 sample had the largest Ti accumulation. When *D. magna* was exposed to different TiO_2_ and Cu^2+^, SOD activity decreased by 31.0% to 64.7% compared with the control experiment (only Cu^2+^). The decrease in SOD activities indicated that both TiO_2_ and Cu^2+^ induced a certain degree of oxidative stress and SOD enzyme inactivation^30^. The nanotoxicity theories were generated by the reactive oxygen species (ROS) and oxidative stress effects^31^. Nanoparticle stress resulting in ROS generation has already been reported by the Dalai groupand could be related to TiO_2_ NP cytotoxicity potential^32^. When *D. magna* was exposed to two foreign materials, SOD activities in the organisms were further deactivated. In addition, SOD activities in the exposed group were evidently lower than Cu^2+^ only, implying that Cu and nano-sized TiO_2_ together are more dangerous than Cu alone in aquatic environments.

Na^+^/K^+^-ATPase indicates the ability of ion transfer in the cell membrane channel. Figure 5b shows the activities of Na^+^/K^+^-ATPase enzyme in *D. magna* under different exposure conditions. Compared with the control group, the Na^+^/K^+^-ATP activities exhibited no significant difference after being exposed only to different TiO_2_. The result is similar to that in C.S. Ramsden’s report, demonstrating that no changes in Na^+^/K^+^-ATPase activity were observed in the brain, gill, or liver tissues of the zebrafish after exposure to TiO_2_ NPs or bulk^33^. Na^+^/K^+^-ATPase enzyme is present at high concentrations in salt-transporting tissues such as intestines and gills, where it maintains the ionic and electrical gradients necessary for transepithelial salt movements. No significant changes in K^+^, Na^+^ and Ca^2+^ concentration were observed in exposure TiO_2_-only conditions, which resulted in the absence of any treatment-related change in Na^+^/K^+^-ATPase activities. When TiO_2_ co-existed with Cu^2+^, the Na^+^/K^+^-ATPase activities in *D. magna* were slightly lower than the treatment with TiO_2_ only. The addition of Cu^2+^ changed the ionic strength of the solution, and Cu^2+^ accumulation in the body inhibited Na^+^ influx and reduced Na^+^/K^+^-ATPase activity in organisms^34^. These results suggest that membrane damage is not the main toxicity to *D. magna* under this research.

### Mechanism of TiO_2_ NSs effects on Cu^2+^ biotoxicity

**Figure 6:**
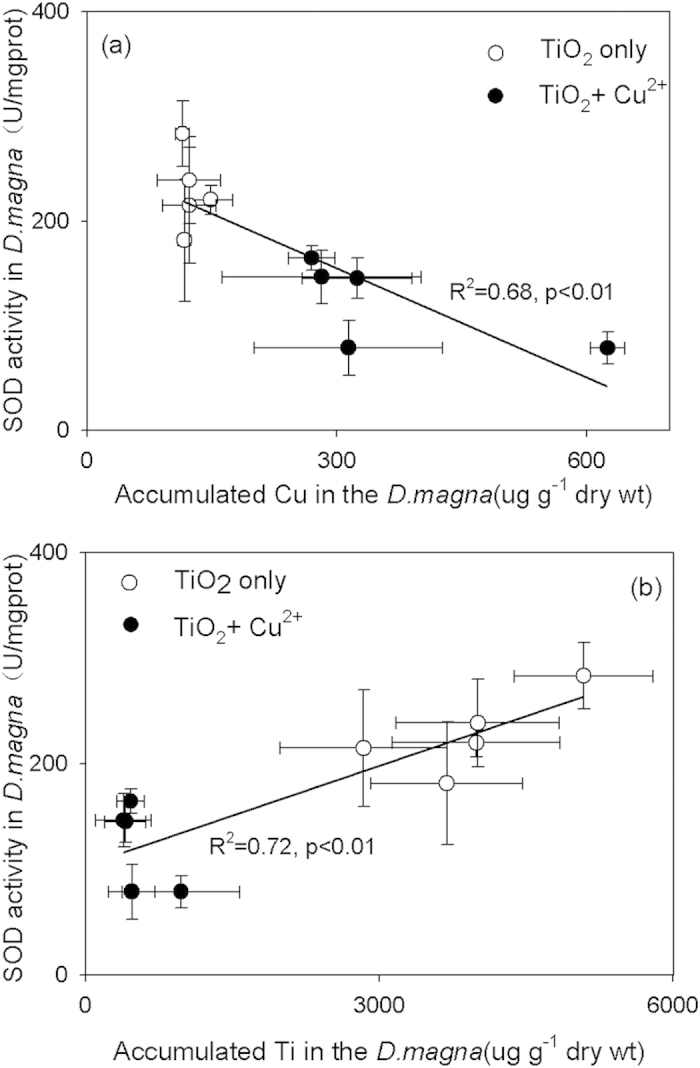
Relationships between SOD activity and accumulated Cu (**a**) and Ti (**b**) in *D. magna* after 48 h exposure to the TiO_2_ samples prepared with varying *R*_F_ in the absence and presence of Cu^2+^. Mean ± standard deviation (n = 3).

In the coexistence system, Cu^2+^ affected the stabilities of TiO_2_ NS suspensions and their ingestion by organisms. The addition of TiO_2_ NSs changed Cu^2+^ uptake and biotoxicity in *D. magna*. As suggested above, the oxidative stress damage instead of membrane damage is the major toxicity. To further investigate the main mechanisms of oxidative stress toxicity, the relationship between superoxide dismutase (SOD) activity and Cu/Ti accumulation was considered when *D. magna* was exposed to Cu^2+^ and different TiO_2_ samples. According to Fig. 6, a linear relationship between SOD activity and Cu/Ti accumulation in *D. magna* (*P* < 0.01, one-way ANOVA) appears. SOD activities decreased with increasing copper accumulation and decreasing Ti accumulation in *D. magna*. These results are related to the physiological effect of Cu and Ti on organisms.

Generally, Cu^2+^ is a hazardous substance to *D. magna* and could produce strong oxidative damage. Cu^2+^-induced cellular toxicity can be explained by the participation of Cu^2+^ in the formation of ROS. Cu^2+^ can be reduced to Cu^+^ in the presence of superoxide (O_2_^−^•), and Cu^+^ is capable of catalyzing the formation of hydroxyl radical (OH•) from hydrogen peroxide (H_2_O_2_)^35^. OH• is a strong oxidizing radical that can practically react with every biological molecule and destroy the antioxidant defense system. SOD enzyme activities in *D. magna* were deactivated with the accumulation of Cu. On the contrary, Ti is the ninth most abundant element in the earth’s crust, and has a certain stimulating and promoting effect on the growth of plants^36^. Its beneficial effects on plants have been known since the 1930s^37^. One mechanism of Ti action is that Ti^4+^/Ti^3+^ participates in the metabolism reaction involved in electron transfer in the redox system^38^. Ti species also involve in activating enzyme activities, such as peroxidase, catalase and nitrate reductase activities in plant tissues^39^. For these reasons, Ti in daphnids possibly maintains higher SOD enzyme activities to help in the scavenging of generated ROS. However, this supposition needs further studies.

In summary, it was found that bioaccumulation and biotoxicity of nanostructured TiO_2_ in *D. magna* was dependent on the percentage of exposed {001} facets. With the co-existence of nanostructured TiO_2_ and Cu^2+^, the percentage of exposed {001} facets influenced on the interaction between TiO_2_ and Cu^2+^ and therefor played an important role on Cu^2+^ bioaccumulation and biotoxicity in *D. magna*. Firstly, Ti bioaccumulation in *D. magna* increased slightly with increasing percentage of {001} facets, and the addition of Cu^2+^ reduced Ti bioaccumulation in organisms due to the aggregation of TiO_2_ induced by adsorbed Cu^2+^. Secondly, TiO_2_ NPs enhanced copper accumulation, whereas the other four TiO_2_ NSs reduced it. Such difference is probably relevant to the different Cu^2+^ adsorption capacities of TiO_2_ with different percentage of exposed {001} facets. Thirdly, five types of TiO_2_ and Cu ingested by *D. magna* produced relatively strong oxidative stress and inhibited SOD enzyme activity, but the membrane damage was not the main toxicity. Moreover, the SOD activities decreased with increasing copper accumulation because of its oxidative toxicity, whereas SOD increased with increasing Ti accumulation in *D. magna* probably because of Ti’s positive physiological effect. In sum, it was confirmed that the co-existence of copper and TiO_2_ is more dangerous than copper alone in aquatic environments. The mechanism of TiO_2_ NSs on copper biotoxicity requires further exploration.

## Methods

### Preparation of TiO_2_ NSs and NPs

TiO_2_ NSs samples were prepared through solvothermal method using Ti(OC_4_H_9_)_4_ and HF solution as precursors^14^,^15^. Briefly, 25 mL of Ti(OC_4_H_9_)_4_ and 3 mL of HF solution (with a concentration of 40 wt.%) were mixed in a dried 100 mL Teflon-lined autoclave, then heated and kept at 180 °C for 24 h. The nominal atomic ratio of F to Ti (***R***_F_) was 1. After the solvothermal reaction, the white precipitates were collected after thorough rinse in ethanol and distilled water thrice, and drying in an oven at 80 °C for 6 h. Four TiO_2_ NSs samples with different percentages of exposed {001} facets were prepared by changing ***R***_F_ (0.67, 1.00, 1.33 and 2.67). Based on the geometric configurations derived from TEM images, the four prepared TiO_2_ NSs samples appear 61%, 71%, 74%, and 78% exposed {001} facets, which were labeled as NS61, NS71, NS74, and NS78, respectively. TiO_2_ NPs with 10% exposed {001} facets were hydrothermally prepared in pure water without HF and labeled as NP10. The preparation details of TiO_2_ NSs and NPs are summarized in Table 1. Finally, TiO_2_ stock suspensions (1 g L^−1^) were prepared by dispersing TiO_2_ NSs or NPs in Milli-Q water using ultrasonic treatment for 30 min (50 W L^−1^, 40 kHz). The stock solution was stored at room temperature before utilization.

### Characterization

Transmission electron microscopy (TEM) analysis was conducted using a JEM-2100F electron microscope (JEOL, Japan) with an accelerating of 200 kV voltage. X-ray diffraction (XRD) (type HZG41B-PC) was used to characterize the crystalline phase and crystallite size of the TiO_2_ samples. Brunauer–Emmett–Teller (BET) specific surface area (*S*_BET_) of the powders was analyzed via nitrogen adsorption in a Micromeritics ASAP 2020 nitrogen adsorption apparatus (USA). All the as-prepared samples were degassed at 180 °C prior to nitrogen adsorption measurements. The BET surface area was determined by a multipoint BET method using adsorption data in the relative pressure (*P/P*_*0*_) range of 0.05 to 0.3. A desorption isotherm was used to determine the pore size distribution via the Barrett–Joyner–Halenda (BJH) method, assuming a cylindrical pore modal.

### Adsorption of Cu^2+^ on TiO_2_

To study the sorption of Cu^2+^ on nano-sized TiO_2_ with different percentages of {001} facets, 1 mg/L nano-sized TiO_2_ suspensions were prepared using the TiO_2_ stocks in SM7 medium respectively. Add Cu^2+^ solution with a known concentration into TiO_2_ suspension and mix rapidly. Tow replicates were set for each treatment. At 5, 30, 60, 120, 240 and 360 min, 5 mL of the mixture was drawn out. The samples were then centrifuged for 5 min at 12,000 rpm using a versatile compact centrifuge (Himac CF 16RX, Hitachi, Tokyo, Japan) to separate particles from the solution. Cu^2+^ concentration in the supernatant was determined through ICP-MS (VG PQ2 TURBO). The adsorption amount of Cu^2+^ on TiO_2_ was determined by calculating the mass difference between before and after adsorptions.

### Model organisms

The *D. magna* used in this study was kept in the laboratory for two years, and were cultured at 23 °C with a light:dark cycle of 16:8 h. The daphnids were cultured in natural water collected from Huo Qi Ying Bridge (116°16’ 732 E, 39°58’ 401 N). The water used was filtered through a 1.2 μm membrane before use. The green alga *Chlamydomonas reinhardtii* was fed to *D. magna* at a density of 1 × 10^5^ to 2 × 10^5^ cells mL^−1^ per day, and the water was replenished every two days. The alga was grown in artificial WC medium^40^ and was collected at its exponential growth stage by centrifugation.

### Acute exposure of *D. magna* to TiO_2_ with or without Cu^2+^

The water used for the exposure experiments was synthetic water, which was simplified Elendt M7 medium (SM7, containing only CaCl_2_, MgSO_4_, K_2_HPO_4_, KH_2_PO_4_, NaHCO_3_, NaNO_3_, Na_2_SiO_3_, H_3_BO_3_, and KCl and without disodium ethylenediaminetetraacetic acid, trace metals, or vitamins). The stocks of five different TiO_2_ were added to 500 mL SM7 with TiO_2_ concentration fixed at 1 mg/L. Two groups of samples were set: one contained TiO_2_ and another contained TiO_2_ and Cu^2+^ fixed at 50 μg/L. The control group comprised 500 mL SM7 and Cu^2+^. Each treatment had three replicates, which contained 50 14-day old *D. magna* (1 individual/10 mL). The *D. magna* were not fed during the exposure time. All the samples were treated under the same conditions. All glassware and exposure chambers were previously acid washed and thoroughly rinsed with distilled water.

### Determination of Ti and Cu bioaccumulation in *D. magna*

At the end of exposure, ten *D. magna* were taken out and rinsed with pure water for three times. They were then placed in a drying oven at 80 °C. These dried *D. magna* were digested in 68% HNO_3_ (Aristar grade) and (NH_4_)_2_SO_4_-H_2_SO_4_ (98%, Aristar grade) solution at 110 °C^41^. The digestion solution was transferred into a volumetric flask with 2% HNO_3_ and diluted for Ti and Cu analysis through ICP-MS. TiO_2_ and Cu accumulation was calculated based on the dry weight of *D. magna* (μg/g dry wt).

### Determination of SOD and Na^+^/K^+^-ATPase activities in *D. magna*

The other twenty exposed *D. magna* were weighed after wiping off the water from their surfaces. Tissues of *D. magna* were homogenized in 0.5 mL sucrose buffer (0.25 M sucrose and 0.1 M Tris-HCl, pH 8.6) by ultrasonication, after which they were centrifuged at a speed of 16000 × g for 20 min. The supernatant fluid was diluted to 1.5 mL using a homogenate. One milliliter of supernatant fluid was used to determine SOD enzyme and Na^+^/K^+^-ATPase activities using commercially available kits (Nanjing Jiancheng Bioengineering Institute, China) according to the manufacturer’s protocol.

SOD is a kind of catalytic enzyme that can convert superoxide into oxygen and hydrogen peroxide to protect cells. SOD activity is assayed using a spectrophotometric method based on inhibition of a superoxide-driven NADH oxidation, which consists of a purely chemical reaction sequence which involves EDTA, Mn(II), mercaptoethanol, and molecular oxygen^42^. Na^+^/K^+^-ATPase can keep a high concentration of K^+^ inside the cell and Na^+^ outside the cell to maintain the balance of osmotic pressure. Na^+^/K^+^-ATPase is assessed based on the amount of inorganic phosphate liberated from hydrolysis of the substrate ATP^43^.

## Additional Information

**How to cite this article**: Liu, L. *et al.* Effects of the interaction between TiO_2_ with different percentages of exposed {001} facets and Cu^2+^ on biotoxicity in *Daphnia magna. Sci. Rep.* **5**, 11121; doi: 10.1038/srep11121 (2015).

## Figures and Tables

**Table 1:** Effects of *R*_F_ on physical properties of TiO_2_.

**No.**	***R***_**F**_	**Percentage of {001}**	**Phase**	**CS (nm)**	***S***_**BET**_**(m**^**2**^**/g)**	**APS (nm)**	**PV (cm**^**3**^**/g)**	**Porosity (%)**
NP10	0	10	A	8.9	156	7.4	0.33	55.0
NS61	0.67	61	A	12.5	128	8.8	0.35	56.5
NS71	1	71	A	13.6	114	16.0	0.52	65.8
NS74	1.33	74	A	15.1	108	19.0	0.53	66.3
NS78	2.67	78	A	17.9	97	20.0	0.56	67.5

A, CS, APS, and PV represent anatase, crystalline size, average pore size and pore volume, respectively.
